# Bitter melon (Momordica charantia) attenuates atherosclerosis in apo-E knock-out mice possibly through reducing triglyceride and anti-inflammation

**DOI:** 10.1186/s12944-018-0896-0

**Published:** 2018-11-06

**Authors:** Yanmei Zeng, Meiping Guan, Chenzhong Li, Lingling Xu, Zhongji Zheng, Jimin Li, Yaoming Xue

**Affiliations:** Department of Endocrinology and Metabolism, Nanfang Hospital, Southern Medical University, No.1838, Guangzhou Avenue, Guangzhou, 510515 Guangdong China

**Keywords:** Bitter melon, Triglyceride, Atherosclerosis, Anti-inflammation

## Abstract

**Background:**

Bitter melon (BM, Momordica charantia) has been accepted as an effective complementary treatment of metabolic disorders such as diabetes, hypertension, dyslipidemia and etc. However it is unclear whether BM can prevent the progression of atherosclerosis. To confirm the effects of BM on atherosclerosis and explore its underlying mechanisms, we design this study.

**Methods:**

Twenty four male apolipoprotein E knock-out (ApoE-/-) mice aged 8 weeks were randomly divided into control group fed with high fat diet (HFD) only and BM group fed with HFD mixed with 1.2%w/w BM. After 16 weeks, body weight, food intake, blood glucose, serum lipids were measured and the atherosclerotic plaque area and its histological composition were analyzed. The expression of vascular cell adhesive molecules and inflammatory cytokines in the aortas were determined using quantitative polymerase chain reaction.

**Results:**

Body weight gain and serum triglycerides (TG) significantly decreased in BM group. BM reduced not only the atherosclerotic plaque area and the contents of collagen fibers in atherosclerotic plaques but also the serum soluble vascular cell adhesion molecule (VCAM)-1 and P-selectin levels, as well as the expressions of monocyte chemoattractant protein (MCP)-1 and interleukin (IL)-6 in aortas.

**Conclusion:**

Our study indicates that dietary BM can attenuate the development of atherosclerosis in ApoeE-/- mice possibly through reducing triglyceride and anti-inflammation mechanism.

## Background

Atherosclerosis is a chronic inflammatory response in arteries characterized by deposition of lipids, recruitment of monocytes, formation of macrophage foam cells, proliferation and migration of vascular smooth muscle cells (VSMCs) and endothelial dysfunction [1, 2]. Abundant evidences showed that Traditional Chinese Medicine (TCM) can present the progression of atherosclerosis via anti-oxidation and anti-inflammation [3–5]. The bioactive components of bitter melon, one of the most common TCMs, have been reported to improve the glucose and lipids metabolism, important risk factors of atherosclerosis, via anti-oxidation and anti-inflammation [6–9]. Furthermore, studies show that BM can regulate metabolism through the activation of peroxisome proliferator-activated receptor (PPAR) and AMP-activated protein kinase (AMPK) [10–16], which may represent protective mechanisms against atherosclerosis via potential anti-inflammation in endothelial cells [17, 18]. Recently the fermented milk-soymilk supplemented with M. charantia (one of the bitter melon ) was reported to have an anti-atherosclerotic activity by increasing superoxide dismutase (SOD) and total antioxidant status (TAS) activity in hyperlipidemic hamsters [19]. However, it is unclear whether BM itself can prevent the progression of atherosclerosis. In the present study, we investigated the effects of BM on the development of atherosclerosis induced by HFD in ApoE-/- mice and explored the potential mechanisms.

## Methods

### Animals and diets

ApoE-/- mice with the C57BL/6 genetic background provided by Joslin Diabetes Center, Harvard Medical School (Boston, MA, USA ) were breeding in a pathogen-free environment with a 12 h light/dark cycle with free access to food and water and performed according to institutional and governmental guidelines. A high-fat diet containing 21.8% fat providing 42% energy and 1.25% cholesterol was purchased from GDLAC (GDLMC, Guangzhou, China). Twenty four male mice aged 8 weeks were randomized into control group (HFD, n=12) fed with high fat diet (HFD) only and BM -treated group (BM, n=12) fed with HFD mixed with BM at a dose of 1.2%w/w for 16 weeks, based on a previous study [20]..

### Analysis of serum metabolic profile

Body weight and food intake of animals were recorded weekly. Intraperitoneal glucose tolerance test (IPGTT), with injection of 20% glucose at a dose of 2g/kg, was administered with tail vein blood at week 14 with One Touch Ultra meter (Lifescan; Johnson & Johnson, USA) at 0, 15, 30, 60 and 120 min. For the measurement of the blood lipids profile, blood was collected from orbital sinus of the animals which already fasting for 8 hours and anesthetized by isoflurane. We centrifuge the blood samples at 1500g 4^o^C for 10min to obtain the serums and store at -80oC. The measurement of the serum levels of TG, TC, LDL, HDL, VLDL are operated on BECKMAN AU-5800 automatic biochemical analyzer with Olympus original reagent, calibration and quality control solution(Olympus, Japan). Serum TG levels were measured by Glycerophosphate oxidase - peroxidase (GPO-PAP) method. Serum TC levels were determined by the enzymatic method, HDL levels were determined by chemical modification method. LDL and VLDL levels were determined by selective lysis enzymatic method.

And the serum soluble adhesion molecules were determined by enzyme-linked immune-sorbent assay (R&D Systems, UK). In addition, aortas and other tissues were collected at the end of the study and quickly frozen in liquid nitrogen and then stored at −80°C for later analysis.

Quantification of atherosclerotic lesion area and measurements of atherosclerotic plaques histological composition

The aortas after opening and fixing in 10% formalin for 36h were stained with Sudan IV to detect the atherosclerotic lesion area and then photographed by a digital camera connected to a dissection microscope. The amount of atherosclerosis lesion was evaluated as the ratio of the atherosclerotic lesion area to the whole aorta area by Image-Pro Plus 6.0.

The aorta fixed in 10% formalin after 24h was paraffin embedded and cross-sectioned to analyze its histological composition. Masson’s trichrome stain kit (Maiwei, Xiamen, China) was used to assess the content of collagen fibers in atherosclerotic plaques and immune-histochemistry incubated with goat anti-macrophage-2 antigen (MAC-2) mouse macrophage or anti-alpha-smooth muscle cell (α-SMA) specific actin polyclonal antibody respectively (Bioss, Beijing, China) to qualify the contents of vascular smooth muscle cells and macrophage cells. All cross-sections were analyzed under an upright microscope (Nikon, Tokyo, Japan) .The amounts of macrophage and smooth muscle cells as well as the collagen fibers were quantified by image-processing software (Image Pro-plus 6.0).

### Determination of Inflammatory cytokines mRNA levels in aortas

The mRNA levels of MCP-1 and IL-6 were measured by SYBR Green Quantitative Real-time PCR. RNA were ed from aorta tissues with E.Z.N.A Total RNA Kit II (Omega, USA) and then reversed to cDNA with PrimeScript TM RT reagent Kit (Takara biotechnology, Dalian, China) under the following conditions: 37°C for 15 min , 85°C for 5 seconds and 4°C forever. Quantitative Real-time PCR was performed with ABI 7500 (ABI, USA) using SYBR premix Ex Taq (Takara biotechnology, Dalian, China) as follows: 1 cycle at 95°C for 30 minutes; 40 cycles at 95°C for 5 seconds and 60°C for 34 seconds; and 1 cycle 95°C for 15 seconds, 60°C for 1 minute and 95°C for 15 seconds. The primers used as follows: GAPDH F5’-GTG AAGCAGGCATCTGAGGG-3’ and GAPDH R5’-CGAAGGTGGAAG AGTGGGAGT-3’; MCP-1 F5’-GCA GCA GGT GTC CCA AAG AA F-3’ and MCP-1 R5’- ATT TAC GGG TCA ACT TCA CAT TCA A -3’; IL-6 F5’-AGG AGA CTT GCC TGG TGA AAA-3’ and IL-6 R5’- AAA GCT GCG CAG AAT GAG ATG-3’. The relative quantification values for the mRNA expressions of MCP-1 and IL-6 were calculated by ΔΔCT .

### Statistical analysis

All data were expressed as mean±SD or mean±SEM. Comparisons of means between the two groups were analyzed by un-paired Student’s *t* test. Levene's Test was used for Equality of Variances. A p value<0.05 was considered statistically significant. All analysis were performed using SPSS version 13.0 for Windows.

## Results

### Effects of BM on body weight, food intake and metabolic profiles

**Fig. 1:**
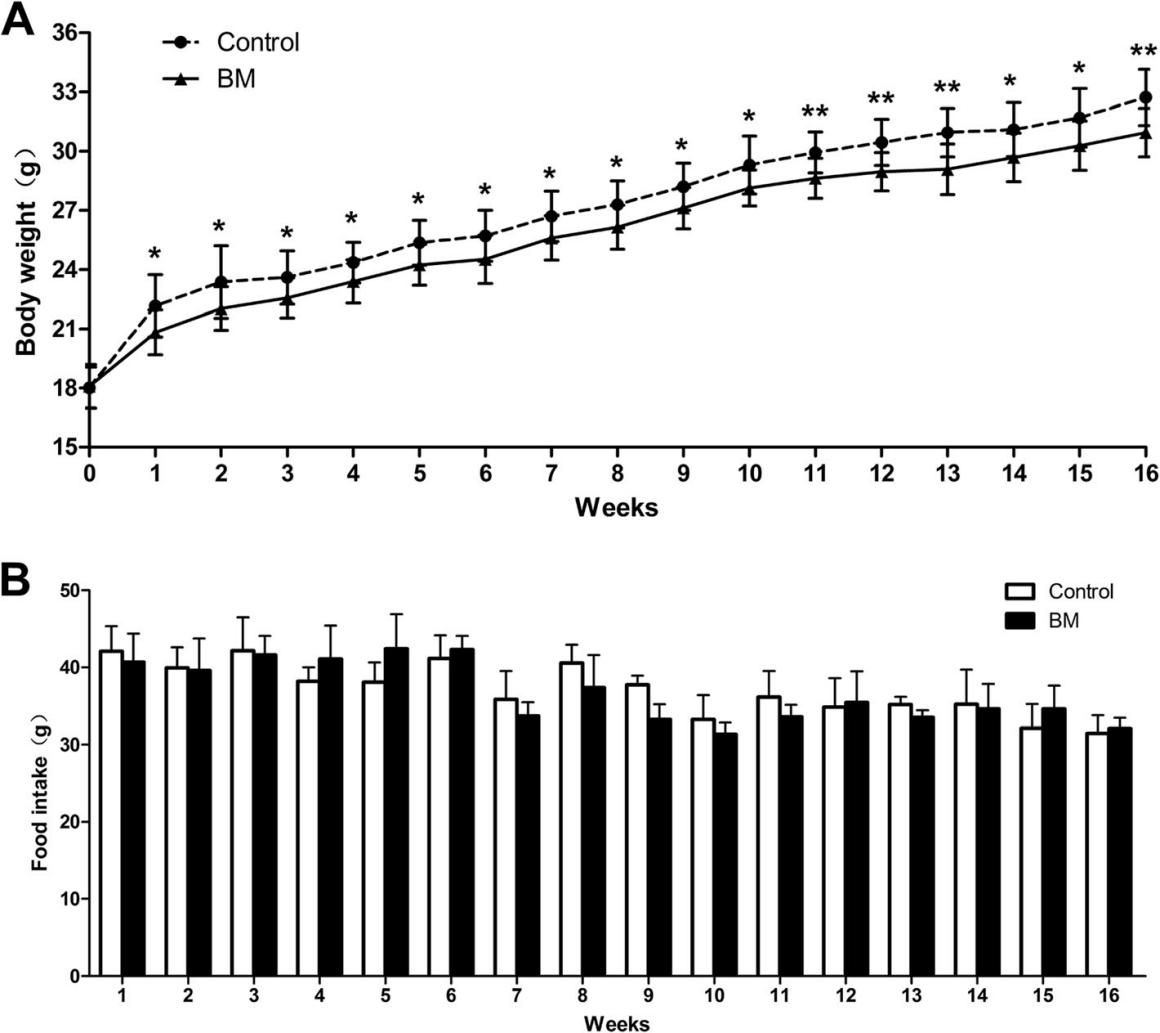
Effects of BM on body weight and food intake in mice. **a** shows the body weight; (**b**) illustrates food intake in the two groups of mice; Data are presented as mean±SD, *n*=8-12 per group, Control vs BM. There is no differences in the body weight and food intake as well as blood glucose levels between the two groups

**Fig. 2:**
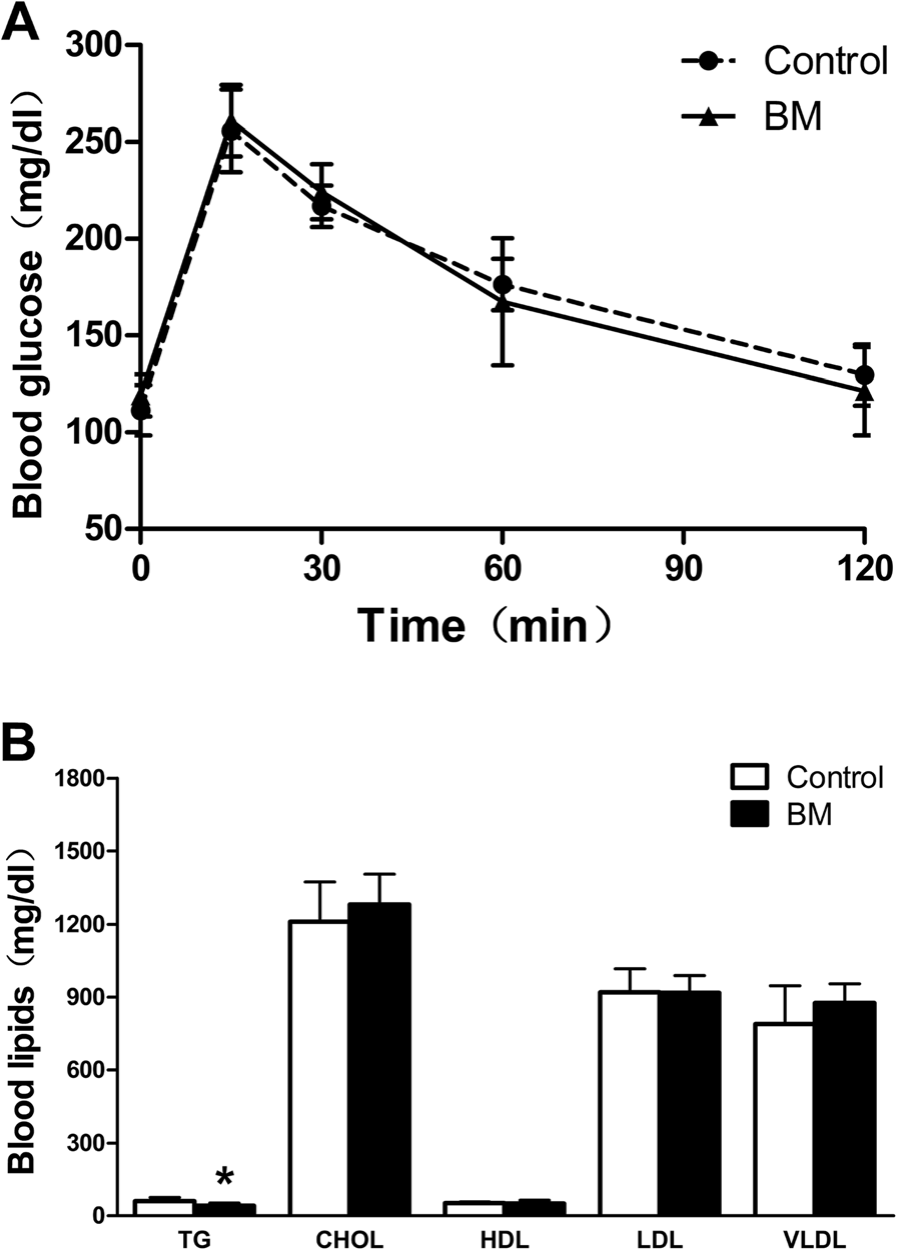
Effects of BM on IPGTT and blood lipids in mice. **a** The blood glucose levels at 0, 15, 30, 60 and 120 min after injection of glucose in the two groups. **b** BM decreased the level of TG in ApoE-/- mice, while with no effect on TC, LDL, HDL and VLDL. Values are mean±SD; n=8 per group; ^*^p<0.05. Control vs BM

There were no differences between the two groups in body weight at baseline (18.02±1.05 and 18.09±1.10 g; HFD vs BM; *n*=12), but after 16 weeks HFD resulted in a significantly increase of body weight in both groups (32.73±1.43 and 30.95±1.23 g; HFD vs BM; *n*=12; Table 1). Compared with the control group, the body weight significantly decreased in BM group from 2 to 16 weeks (*p *<0.05, Fig. 1a).There was no significant difference in food intake and blood glucose levels at 0, 15, 30, 60 and 120 min between the two groups (Fig. 1b and Fig.2a). BM decreased the serum TG level significantly (41.04±11.72 vs 60.73±15.62 mg/dl; p=0.013; Table 1 and Fig. 2b), while with no effect on TC, LDL ,VLDL and HDL (*p* >0.05; Table 1 and Fig. 2b).

**Table 1:** Data on body weight, food intake, fasting blood glucose, and blood lipid profile

	Control	BM	*P* value
Daily Food intake(g/w)	37.13±3.42	36.79±3.62	0.79(NS)
Body weight(g) pretrement	18.02±1.05	18.09±1.10	0.87 (NS)
Body weight(g)	32.73±1.43	30.95±1.23^b^	0.004
Fasting blood-glucose(mg/dl)	109.58±10.65	119.03±10.82	0.10 (NS)
TC(mg/dl)	1211.02±163.79	1281.16±123.93	0.35(NS)
TG(mg/dl)	60.73±15.62	41.04±11.72^a^	0.013
HDL (mg/dl)	52.78±5.25	50.60±14.32	0.69(NS)
LDL(mg/dl)	919.61±96.49	917.92±70.75	0.97(NS)
VLDL(mg/dl)	789.24±157.62	876.07±78.52	0.19(NS)

Table1 *TG* as triglycerides, *HDL* as high-density lipoprotein, *LDL* as low-density lipoprotein and *VLDL* as very low-density lipoprotein. Values are expressed as mean±SD; *n*=8-12 per group; ^a^p<0.05, ^b^p<0.01; Control versus BM

### BM reduced the area of atherosclerotic lesion in ApoE-/- mice

**Fig. 3:**
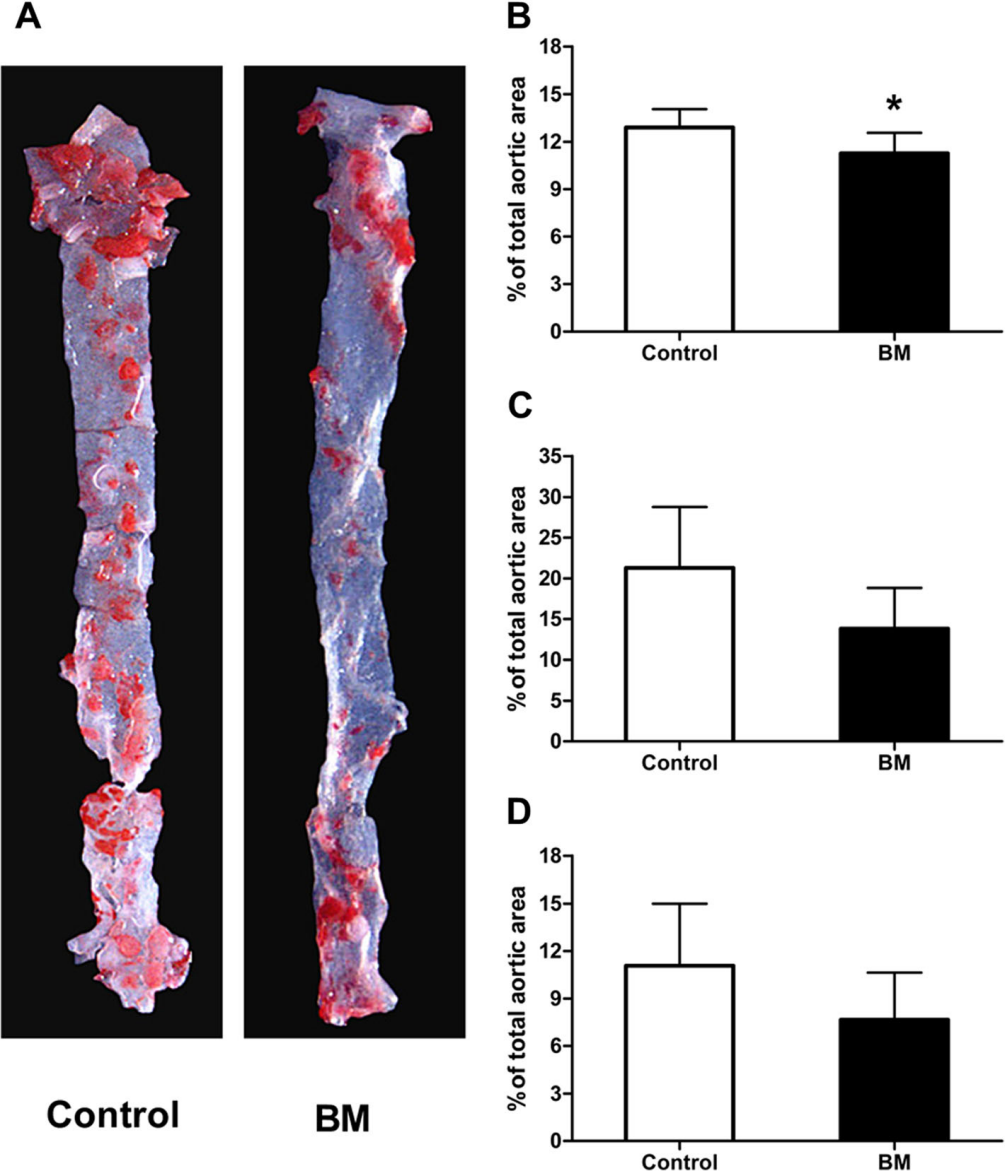
BM reduced atherosclerotic lesion areas in aortic sections of ApoE–/– mice. **a** Quantitative analysis of atherosclerotic lesion area was measured on the intimal surface of (**b**) aorta (**c**) aortic arch and (**d**) abdominal aorta of mice in the two groups. Data are mean±SD, *n*=6. ^*^p<0.05 versus control

The atherosclerotic plaques were showed by Sudan IV staining as Fig. 3a. In ApoE-/- mice fed with HFD, there were fewer atherosclerotic plaques of the entire aorta in BM group than in control group (12.91±1.15 vs 11.25±1.31 %, *p*=0.042; Fig. 3b). We found decreasing tendencies exist in the atherosclerotic plaque area of the aortic arch and abdominal aorta under the treatment of BM , although with no statistical significant (*p*=0.068 and *p*=0.12; Fig. 3c, d).

### Effects of BM on the histological composition of the atherosclerotic plaques

**Fig. 4:**
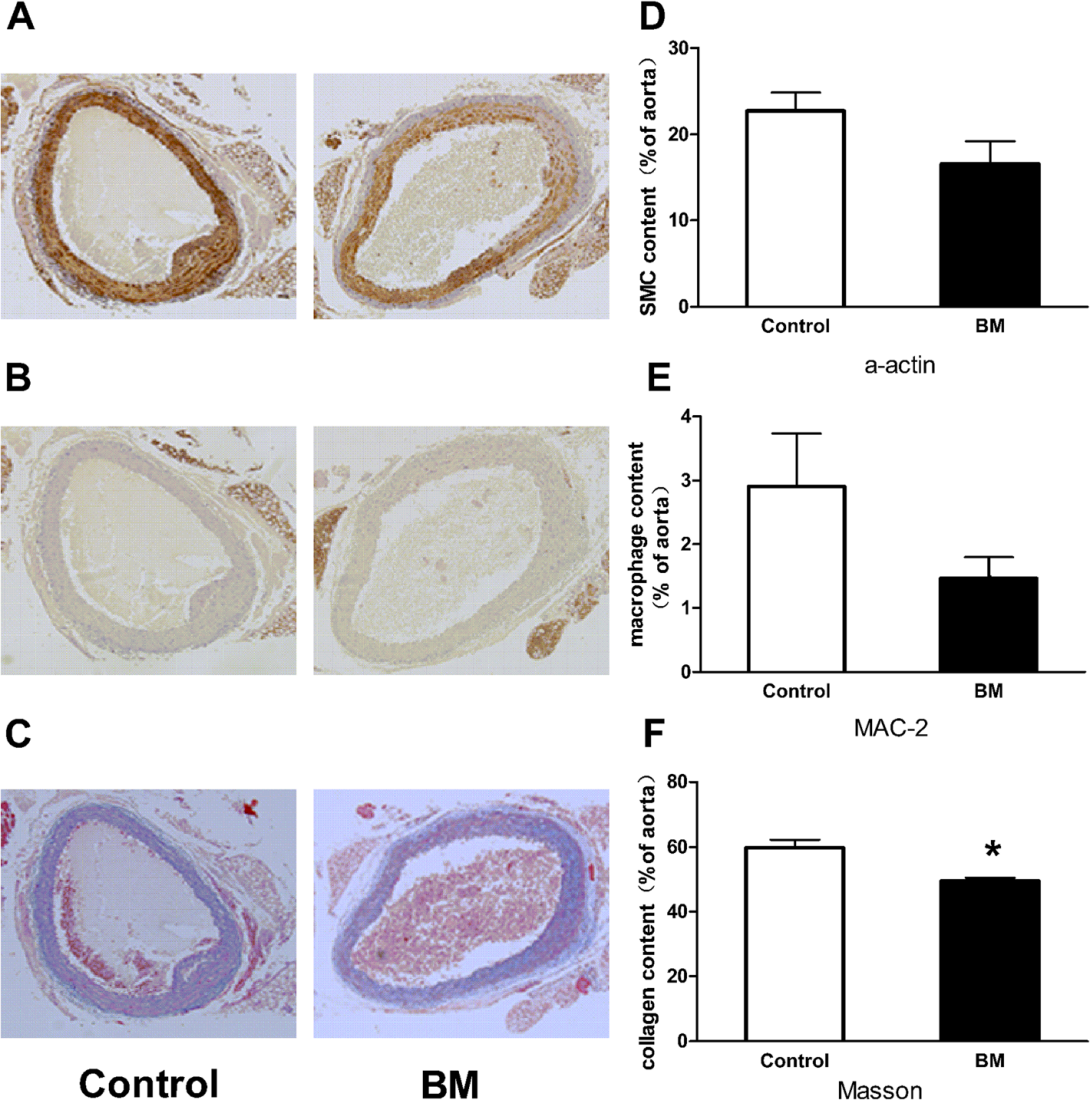
Effects of BM on expression of α-SMA, MAC-2 and collagen fibers in atherosclerotic plaques. Representative images of smooth muscle cells (**a**), macrophages (**b**) and collagen fibers (**c**) in atherosclerotic plaques ( n=6 for each group). Quantitative analysis of smooth muscle cells (**d**), macrophages (**e**) and collagen fibers (**f**) in atherosclerotic area (magnification ×100; n=6 for each group). Values are mean ± SEM; ^*^p<0.05; Control vs BM

The histological composition of atherosclerotic plaques containing vascular smooth muscle cells, macrophages and collagen fibers were analyzed by immunohistochemistry and Masson’s trichrome staining (Fig. 4a, b, c). The vascular smooth muscle cells and macrophages expressed in aortic plaques tended to decrease in the BM group compared with the control group, although there was no statistical difference (*p*=0.097 and *p*=0.134; Fig. 4d, e). There were significantly less collagen fibers in aortic plaques in BM group than in control group (49.48±2.54 vs 59.83±5.82 um^2^; *p*=0.03; Fig. 4f).

BM decreased the serum sVCAM-1 and sP-selectin levels and inhibited the expressions of MCP-1 and IL-6 in aortas

**Fig. 5:**
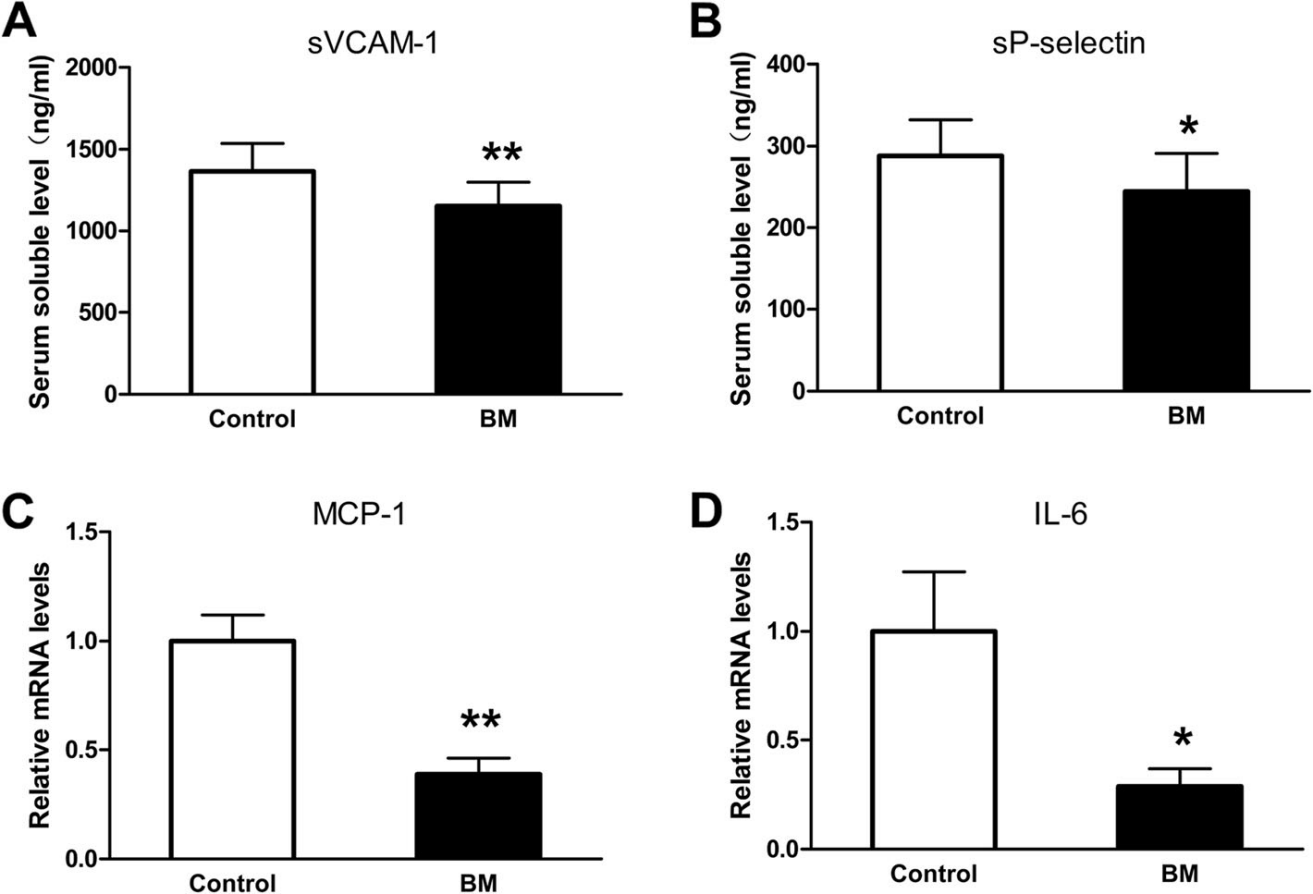
BM decreased the serum soluble adhesive molecules and the expression of inflammatory cytokines in aortas. **a**-**b** Serum concentrations of soluble vascular cell adhesion molecules (VCAM)-1 and P-selectin (*n*=10 each). Values are mean±SD; ^*^p<0.05, ^**^p<0.01; Control vs BM. **c**-**d** mRNA expression levels, Monocyte chemotactic protein (MCP)-1 and interleukin (IL)-6 measured in aortas (*n*=6 each). Values are mean±SEM; ^*^p<0.05, ^**^p<0.01; Control vs BM

The serum levels of adhesion molecules and the expression of inflammatory cytokines in aortas were determined at the end of the study. The serum soluble VCAM-1 and P-selectin significantly decreased in the BM group compared with the control group (1151.92±142.90 vs 1365.18±170.26 mg/dl, p=0.008; 288±44.46 vs 244.43±46.67 mg/dl, *p*=0047; Fig. 5a, b). BM also significantly reduced the expression of MCP-1 and IL-6 in aorta tissues (*p*=0.002 and *p*=0.048; Fig. 5c, d).

## Discussion

In this study, BM was confirmed to reduce the area of atherosclerotic lesion in aortas as well as the content of collagen fibers, smooth muscle cells (SMC) and macrophages in the plaques. Furthermore, we found that BM helps to resist the body weight gain induced by HFD in ApoE-/- mice with no effect on food intake, which is consistent with the previous reports [20]. We observed that the TG, a marker for atherogenic lipoproteins [21], obviously decreased after treating with BM. we also find out the reduction of pro-inflammatory cytokines and cellular adhesion molecules, which play an important role in inflammation of cardiovascular disease, when treated with bitter melon. It has been reported that the concentration of hepatic TG and TC can be decreased by dietary BM in rats [22–24]. In addition, we observed that serum soluble VCAM-1 and P-selectin levels, which play a major role in the initiation of atherosclerosis, were reduced and the expressions of MCP-1 and IL-6 in aortas implicated in mediating the pathogenesis of atherosclerosis were decreased by BM treatment [25–27]. Previous researches suggest that there was a strong and independent association between TG and atherosclerosis. Furthermore, triglycerides are an independent predictor of endothelial function which associated with atherosclerosis. It has been reported that raising-triglyceride increase clinical cardiovascular endpoints but lowering circulating triglyceride levels may improve endothelial function, leading to a decrease in cardiovascular events [28–30]. It has been reported that TG may also stimulate atherogenesis by producing proinflammatory cytokines, fibrinogen and coagulation factors. Therefore our data in this study implicated that BM alleviated the pathogenesis of atherosclerosis possibly through many mechanisms including triglyceride- decreasing, anti-inflammation and endothelial function improvement.

## Conclusion

In summary, our study shows that oral administration of BM can prevent the progression of atherosclerosis and alter the composition of the atherosclerotic plaques possibly through reducing serum TG and potential anti-inflammation. These findings suggest that BM as a nutritional food may plays an important role in CVD protection.

## Abbreviations

AMPK: AMP-Activated protein kinase
ApoE-/-: Apolipoprotein E knock-out
BM: Bitter melon
GPO-PAP: Glycerophosphate oxidase - peroxidase
HDL: High-density lipoprotein cholesterol
HFD: High fat diet
IL-6: Interleukin-6
IPGTT: Intraperitoneal glucose tolerance test
LDL: low-density lipoprotein cholesterol
MAC-2: Anti-macrophage-2 antigen
MCP: Monocyte chemoattractant protein
PPAR: Peroxisome proliferator-activated receptor
SOD: Superoxide dismutase
TAS: Total antioxidant status
TC: Total cholesterol
TCM: Traditional chinese medicine
TG: Triglycerides
VCAM: Vascular cell adhesion molecule
VLDL: Very low-density lipoprotein cholesterol
VSMCs: Vascular smooth muscle cells
α-SMA: Anti-alpha-smooth muscle cell
